# Smoking Cessation in Indigenous Populations of Australia, New Zealand, Canada, and the United States: Elements of Effective Interventions

**DOI:** 10.3390/ijerph8020388

**Published:** 2011-01-31

**Authors:** Michelle DiGiacomo, Patricia M. Davidson, Penelope A. Abbott, Joyce Davison, Louise Moore, Sandra C. Thompson

**Affiliations:** 1 Centre for Cardiovascular and Chronic Care, University of Technology Sydney, Level 7, 235-253 Jones Street (P.O. Box 123), Broadway, NSW 2007, Australia; E-Mail: PatriciaMary.Davidson@uts.edu.au (P.M.D.); 2 Curtin Health Innovation Research Institute (CHIRI), Curtin University, GPO Box U1987 Perth, Western Australia, 6845, Australia; 3 Aboriginal Medical Service Western Sydney, 2 Palmerston Road, Mt. Druitt, New South Wales, 2770, Australia; E-Mails: pennyab@amsws.org.au (P.A.A.); joyce@amsws.org.au (J.D.); louise@amsws.org.au (L.M.); 4 Department of General Practice, School of Medicine, University of Western Sydney, Locked Bag 1797, Penrith South DC, New South Wales, 1797, Australia; 5 Combined Universities Centre for Rural Health, University of Western Australia, P.O. Box 109, Geraldton, Western Australia, 6531, Australia; E-Mail: sandra.thompson@cucrh.uwa.edu.au

**Keywords:** tobacco, smoking cessation, indigenous, interventions

## Abstract

Indigenous people throughout the world suffer a higher burden of disease than their non-indigenous counterparts contributing to disproportionate rates of disability. A significant proportion of this disability can be attributed to the adverse effects of smoking. In this paper, we aimed to identify and discuss the key elements of individual-level smoking cessation interventions in indigenous people worldwide. An integrative review of published peer-reviewed literature was conducted. Literature on smoking cessation interventions in indigenous people was identified via search of electronic databases. Documents were selected for review if they were published in a peer-reviewed journal, written in English, published from 1990–2010, and documented an individual-level intervention to assist indigenous people to quit smoking. Studies that met inclusion criteria were limited to Australia, New Zealand, Canada, and the USA, despite seeking representation from other indigenous populations. Few interventions tailored for indigenous populations were identified and the level of detail included in evaluation reports was variable. Features associated with successful interventions were integrated, flexible, community-based approaches that addressed known barriers and facilitators to quitting smoking. More tailored and targeted approaches to smoking cessation interventions for indigenous populations are required. The complexity of achieving smoking cessation is underscored as is the need to collaboratively develop interventions that are acceptable and appropriate to local populations.

## 1. Introduction: Tobacco-Smoking: A Global Health Issue

Tobacco has been referred to as ‘a global agent of death’ because it kills more than five million people throughout the world each year [1]. Smoking is a crucial modifiable risk factor for cardiovascular disease (CVD) as well as five other leading causes of death worldwide; namely cerebrovascular disease, lower respiratory infections, chronic obstructive pulmonary disease (COPD), tuberculosis, and respiratory tract cancers [2]. Indigenous peoples throughout the world suffer more health disadvantage, disability, reduced quality of life, and higher mortality than non-indigenous people [3,4]. Historical, social, political, and cultural factors and racism contribute to these disparities [4–8] which are exacerbated by limited access to appropriate care and resources [9]. The health disparities between indigenous and non-indigenous residents of developed nations, where resources are plentiful and overall wealth, access, and quality life in the general population are consistently improving, are glaring [4]—smoking contributes significantly to this disease burden.

While rates of smoking in non-indigenous people in Australia, New Zealand, Canada, and the United States (US) have markedly declined over the past thirty years, the same is not true for their Indigenous populations [10]. Smoking rates in these Indigenous populations far exceed those of their non-indigenous counterparts (Table 1), indicating that tobacco control strategies have not been universally effective. Reasons for this are complex and likely include issues of access and appropriateness of services and support, reflecting systemic barriers to improving the health of indigenous peoples. Additional factors contributing to continued high prevalence of smoking in indigenous populations include the normalization of smoking in many communities, the historical role of tobacco, beginning to smoke at an early age, living with smokers, a history of colonization and dispossession [10], and variable acculturation, which contribute to low socio-economic status, low levels of education, and high unemployment [11]. Evidence of efficacy of smoking cessation support exists for other populations, however, evaluations of programs targeted towards indigenous populations are less abundant [12]. Given the variable impact of proven treatments across consumers, strategies should be adapted to local contexts and tailored to individual preferences and needs [2]. Despite the heterogeneity of the world’s indigenous populations, common inferior health and socioeconomic status signal the urgency for effective solutions to be shared.

## 2. Considerations in Indigenous Research

Evaluations of smoking cessation interventions in indigenous people are sparse. Ivers’ review of tobacco programs in Australia for Aboriginal and Torres Strait Islander people showed only four published evaluations of tobacco interventions from 1980–2001, none of which described an individual-level intervention or measured or demonstrated an effect on cessation rates [24]. More recently, Power *et al.* identified a further eleven published reports of individual, community, and legislative-level interventions in Australia between 2001–2007 [25]. Although Power *et al.* concluded that cessation strategies targeting individuals, such as NRT and/or counseling, are likely to aid Indigenous Australians to quit [25], detailed analysis of intervention components was beyond the scope of the review. Given the limited information available, strategies used in non-indigenous populations are often applied to interventions for indigenous people, potentially not meeting the needs of these groups [26].

Multi-level tobacco control strategies, including population and individual approaches, are necessary to address the barriers facing indigenous populations [2,27,28]. When developing smoking cessation approaches targeting individuals, it is important to investigate elements of interventions to replicate in practice settings and inform future interventions. This paper aims to describe, in detail, recent research on individual-level smoking cessation interventions. As the applicability and relevance of some research methods to indigenous populations has been challenged, this review chose not to constrain the information available through the limited focus of a systematic review method, but rather to use an integrative approach suitable for capturing process and contextual information from studies using diverse methodologies [29].

## 3. Methods

The integrative review entailed electronic searches of Medline, PsychInfo, CINAHL, Indigenous Australia, APAIS-ATSIS, ATSIHealth, the Australian Indigenous Health Infonet, and Cochrane databases. The search of the databases used a combination of MeSH headings and keywords and was conducted with the assistance of a health librarian in July 2010. The following search terms were used: smoking cessation, smok*, nicotine, cigarette, tobacco, tobacco use cessation, tobacco use disorder, oceanic ancestry group, health services, indigenous, aborigin*, native, health intervention, health promotion, and patient education. Reference lists of obtained articles were also searched for relevant material.

Documents were selected for review if they were published in a peer-reviewed journal, written in English, published from 1990–2010, and documented an individual-level intervention to assist indigenous people to quit smoking. For the purposes of this review, we define an individual-level intervention as one that involves an interpersonal interaction, between a health professional or facilitator and an individual. The interaction can occur via telephone or face-to-face and may involve either one-to-one or group formats. Articles that did not describe the individual-level intervention portion of a multi-component program and those that did not report collective outcomes of indigenous participants separately from non-indigenous participants were excluded.

Titles and abstracts were assessed for relevance independently by two reviewers. Relevant studies were assessed for inclusion independently, with disagreements resolved through discussion. Data were extracted from primary sources on all aspects of the interventions and tabled. If facets of interventions were not included in the documentation, the interventions were considered not to have those characteristics. In addition, two reviewers separately categorized a list of elements of interventions mentioned using a general inductive approach [30]. These categories emerged organically from the data and were discussed within the research team to check consistency of categories. When overlap of categories was low, further discussion assisted in developing a more robust set of categories [30].

## 4. Results and Discussion

**Figure 1.**
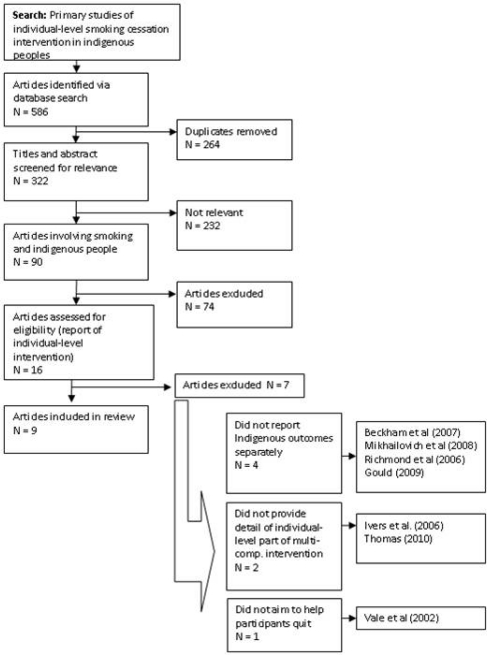
Literature retrieval and selection process.

Database and hand-searching yielded 586 articles (Figure 1). Following exclusion of articles due to duplication (n = 264), 322 abstracts and titles were assessed for relevance to smoking in indigenous populations. Of the remaining 90 articles, 16 reported on primary studies of individual-level smoking cessation interventions in indigenous people. Among these, four studies were excluded because they did not report outcomes for indigenous participants separately from non-indigenous participants. Two studies of multi-component interventions were excluded because they did not report in any detail on the individual-level component of the intervention. One study was excluded because it aimed to encourage participants to reduce smoking rather than quit. The remaining nine articles were reviewed.

Although the research team intended to review studies of various indigenous populations throughout the world, the review included only nine articles from four developed countries; Australia (4), the USA (3), Canada (1), and New Zealand (1) (Table 2). Six of the articles depicted interventions whereby participation was on a one-to-one basis while the remaining three articles depicted small group format interventions. Interventions incorporated counseling or support both with (8) and without (3) use of pharmacological cessation aids (two studies included non-pharmacotherapy comparison groups). One study was a randomized controlled trial.

### 4.1. Pharmacotherapy and Individual Counseling

#### 4.1.1. Bupropion & face-to-face and/or telephone counseling

In a randomised, placebo-controlled, double blind, parallel group study with 12-month follow-up, Holt *et al.* [31] assessed whether bupropion was an effective smoking cessation treatment in the Maori population in New Zealand. Participants were 134 Maori smokers aged 16–70 years who smoked more than 10 cigarettes per day (72% women; mean age 40.5 years) recruited through Maori health networks. A Maori research nurse gave participants a blinded medication pack (seven week supply of bupropion (150 mg once daily for 3 days, then 150 mg twice daily for 7 weeks or placebo). Participants then set a quit date for 7–14 days later. Baseline demographics, nicotine dependence, weight, and carbon monoxide (CO) levels were measured. Participants received a motivational phone call 1 day prior to and 3 days following their quit date. Six clinic visits were scheduled for the next 12 months to assess smoking status and adverse events, measure CO, and provide counselling tailored to individual needs.

The main outcome measures in this study were continued abstinence from smoking at 3 and 12 months. Continued abstinence was better for the subjects allocated to bupropion at all time points; 44.3 percent compared to 17.4 percent at three months and 21.6 percent and 10.9 percent at 12 months. The authors concluded that a community-based program using bupropion combined with counselling is an effective and safe treatment for smoking cessation in the Maori population in New Zealand. Although it was stated that the study involved Maori health providers and was based on principles of cultural safety, additional detail regarding these components was not described.

#### 4.1.2. Nicotine replacement therapy and face-to-face counseling

Ivers assessed use of free nicotine replacement therapy (NRT) patches by Indigenous Australians in the Northern Territory when offered a brief intervention for smoking cessation [32]. Participants self-selected into either a brief intervention (BI)-only group or a BI with NRT group (BI + NRT). The five-minute brief intervention (BI) was based in community health centres, although not specified as Aboriginal Community Controlled Health Services (ACCHS), and involved being given advice on quitting, being shown a flip-chart about tobacco and readiness to quit, and being offered a pamphlet. The researcher and a local Indigenous research assistant conducted the baseline visit in local language, if necessary. Those who opted to use NRT received instructions for use and a one-week supply of graded 24-hour patches without cost. They were asked to return to the health centre for the additional nine weeks of patches.

A six-month follow-up questionnaire assessed the number of patches used, changes in smoking behaviour (point prevalence of smoking status validated by CO test), attitudes to tobacco use, side effects, and barriers to using patches. Of the 111 participants (60 male; 51 female), 40 selected into the BI + NRT and 71 chose the BI-only group. At follow-up, no participant had completed a full course of patches. The average number of patches used was five, but ranged from 0–49 patches. Six participants (15%) reported that they had quit smoking in the patches group (10% were CO validated) and 1 participant (1%) quit in BI-only group. The majority of the remaining participants reported cutting down their smoking.

Ivers *et al.* [32] noted that self-selection into the intervention arm precluded direct comparison of the two groups. Sharing of patches was reported and many participants did not return to collect additional patches. Some participants in the patches group were less willing to make another quit attempt, potentially related to side effects or the perception that the patches were ineffectual. Authors concluded that using nicotine patches may be useful for a small number of Indigenous people who want to quit, but design and delivery of interventions must consider intervention intensity, adherence, and the perceived normality of smoking in the community [32].

Also in Australia, DiGiacomo *et al.* reported a high intensity smoking cessation program at an urban ACCHS [33]. The intervention consisted of weekly cessation counselling sessions (with a non-indigenous health professional) and dispensation of free NRT patches (1 box/week) following a cardiovascular screening and spirometry test administered by Aboriginal Health Workers (AHWs). Nicotine dependency, smoking behaviour, and contextual information regarding family, work, living situation, and health status was discussed. Of the 32 clients who made quit attempts, 3 were abstinent at six months (9%). The majority of clients reported stressful events as causing relapse, leading the authors to conclude that stress management strategies should be incorporated into smoking cessation interventions for Aboriginal Australians.

#### 4.1.3. Nicotine Replacement Therapy and telephone counseling

Maher *et al.* [34] assessed smoking quit rates and satisfaction with the Washington State tobacco quitline (QL). American Indian and Alaskan Native (AI/AN) people comprised 8 percent of the sample (N = 101). The intervention was comprised of at least one phone call with a QL counsellor who had received mutlicultural sensitivity and motivational interviewing training. The counsellor linked participants to local community resources, mailed a quit kit with self-help materials, and encouraged them to proactively call the QL whenever support was needed. Individuals who were uninsured, pregnant, enrolled in Medicaid or the Indian Health Service, or were aged 18–29, and willing to set a quit date within 30 days, received eight weeks of free NRT and four additional counsellor-initiated calls for a portion of the study. The number of AI/AN people that received this additional support was not stated.

Telephone surveys assessed callers’ quit status and satisfaction with the service. The 7-day quit rate (self-reported smoking ‘not at all’ and quit date at least seven days prior) at the 3-month follow-up was 31% for all participants and 35% for AI/AN participants. Satisfaction levels with the QL service were high with AI/AN participants reporting overall satisfaction with the programme (93%), likelihood of suggesting QL to others (98%), and satisfaction with the QL counsellor (97%).

Although this intervention appeared successful for about a third of survey respondents, the 7-day self-reported quit rate does not reflect continuous abstinence during the previous three months. However, a key strength of this study was seeking feedback from participants regarding their experiences with the QL.

In a second QL evaluation, Boles *et al.* [35] examined the acceptability and effectiveness of a state-wide tobacco QL for AI/AN in Alaska, compared to non-AI/AN residents. Individuals aged 18 and older who called the Alaska QL for the first time and set a quit date were eligible for proactive follow-up counselling calls and free NRT. The services offered by the Alaska QL were based on a Mayo Clinic protocol and consisted of tobacco use assessment, treatment planning based on stage of readiness to change, up to eight proactive follow-up counselling calls, a quit kit, and free NRT patches. The QL had a single Alaska Native nurse who was available to speak with Alaska Native callers, if requested. No data was presented on number of times this nurse was requested nor was there information regarding availability of this nurse. The QL was a free service staffed by trained nurses 24 hours a day, seven days a week. Three months following initial contact, telephone surveys assessed quit status and satisfaction and cultural appropriateness of the QL.

As in Maher, the 7-day point prevalence quit rate was used. The 112 AI/AN participants comprised 10 percent of the sample and had a quit rate of 22.2 percent at three months compared to non-AI/AN’s 40.7 percent. Thirteen AI/AN participants (15.3%) indicated they would have preferred to talk to an Alaska Native nurse, indicating that this person was not available at all times. Three AI/AN participants (3.5%) thought the questions were too personal; sixteen (18.8%) thought the question pace was too fast; and four (4.7%) responded “no” when asked whether the QL is appropriate for Alaska Native people. Satisfaction levels were comparable to Maher, although style of delivery was highlighted as problematic for some participants, signalling the importance of asking these types of questions.

### 4.2. Individual Counseling Only: Telephone or Face-to-Face Brief Intervention

Hayward *et al.* [36] assessed QLs, without provision of pharmacotherapy, in Aboriginal and non-Aboriginal Canadian smokers. Participants (n = 7082) were first time callers, age 18 and over, who called the QL during a 4-year period, and completed a six-month evaluation. As part of the QL service, participants received basic information and advice, motivational counselling based on scientific protocols, and mailed materials. Proactive services were offered based on commitment to quit within a given timeframe. Demographic characteristics, smoking behaviours, and actions taken toward quitting were recorded at intake and 6-month follow-up. Satisfaction with the service was assessed by whether participants would refer a friend. Use and satisfaction of the service and cessation rates of Aboriginal participants were comparable with non-Aboriginal participants. The lower cessation rates as compared to Maher [34] and Boles [35] may involve the lack of NRT provided, longer duration to follow-up, or lack of cultural tailoring.

Overall, QLs appeared to be effective and acceptable forms of intervention in the three studied Indigenous populations in North America. NRT was provided to at least some participants in two of the QL interventions [34,35], although information was not provided regarding implementation or impact of this component. Superficially, quit rates were higher in the programs that provided NRT, although differences in measurement time preclude direct comparison. While two of the interventions incorporated culturally sensitive service delivery [34,35], the other concluded that even without this tailoring, the intervention was successful in a proportion of its Aboriginal participants [36].

Although satisfaction with QLs represented perceptions of those who did use these telephone services, individuals who were not willing to call a QL were not assessed. Efforts to culturally tailor QL interventions were made in Boles *et al.* [35] and Maher *et al.* [34] who then evaluated satisfaction with the service and its delivery. Maher *et al.*’s [34] approach provided counselors with cultural awareness and sensitivity training within a multicultural context, including, but not exclusive to Indigenous cultures. Boles *et al.* [35] reported having an AI/AN counsellor available, if requested. It is assumed that this person was not available all hours of every day, despite the QL’s constant availability. Some respondents specifically reported that they would have preferred to speak with an AI/AN counsellor, indicating that one was not requested, offered, or available for all participants. Feedback from some participants dissatisfied with intervention delivery is consistent with guidelines for providing counselling services to Alaska Native people which promote avoiding directive advice and fast-paced delivery of interventions [37]. Maher *et al.* [34], with the highest cessation rate of the QL interventions, reported a very high participant satisfaction rate, potentially reflective of the cultural competence training of counsellors. Alternatively, differences in satisfaction and cessation rates may be attributable to the heterogeneity of the Aboriginal populations studied, suggesting that tailoring strategies should be reflective of the targeted community rather than generalising based on indigenous status [35].

The 1 percent validated quit rate of the brief intervention-only group (control) in Ivers *et al.* [32] demonstrated that a low intensity intervention without a pharmacological aide was not effective in this group. The only other counselling-only intervention study included in this review, reported a 10% prolonged abstinence rate at six months [36], although, for some participants, it may have been higher intensity.

### 4.3. Group Interventions: Support Group/Course and Nicotine Replacement Therapy

Three of the smoking cessation interventions used group formats labelled as support group [38], short course [39], and behaviour modification classes [40]; the latter two implying an education component based on established smoking cessation programs designed by state and national health organisations. Hensel [40] assessed efficacy of a cessation program in Alaskan Native people consisting of four group counselling/behaviour modification sessions over a 2-week period or 7 sessions over a 6-week period. Content was based on American Lung Association (*Freedom from Smoking*) and American Cancer Society (*Fresh Start*) programs. Participants had a physical examination upon commencement during which NRT was prescribed. A physician or pharmacist attended the group sessions to discuss NRT. Demographics, smoking activity, use of NRT, and smoking status were recorded at four time points, however, smoking cessation measurement was unclear. Although ethnicity was not stated, one facilitator was an employee of the Alaska Native Medical Center where the intervention was based. One hundred and ninety-three participants (31%) continued until at least the 3-month follow-up. Participation decreased with successive follow-ups as did cessation rates. Twenty-two participants (12%) did not use any patches. Cessation rates were 31%, 30%, 24%, 21% at 3, 6, 9, and 12 month follow-ups, respectively. Results were comparable to programs in other populations and the program was demonstrated to be cost effective.

Mark *et al.* conducted quit smoking support/information groups (n = 22) with 115 Indigenous people living in New South Wales, Australia [38]. Groups were held for 2 hours per week for 6 weeks (later reduced to 4 weeks based on participant feedback). Participants had the option of receiving 3 weeks of free NRT and were encouraged to purchase a further 5 weeks. The intervention was AHW-facilitated, used culturally-specific resources, had the option of a men-only group, provided transport, and featured discussions on NRT, pros and cons of smoking, and barriers to quitting. Most participants (n = 94; 82%) made a quit attempt using NRT. Of nearly a third (31%) of participants who completed the 4 or 6-week program, 16 (44%) reported continued abstinence indicating a 14% quit rate at program end while others reported having cut down. At the 3-month follow-up, 6% reported abstinence. As a result of the groups, most participants were more confident to make another quit attempt.

Adams *et al.* conducted a group-format intervention based in a rural ACCHS in Australia, where a trained community health nurse and AHW facilitators conducted short courses with QL support [39]. The course [41,42] entailed 2 half-day classes with group discussion on understanding smoking behaviour, preparing to quit, and the quitting experience. Participants received a QL course booklet, behaviour modification items, and the opportunity to register for QL telephone support with access to subsidised NRT or bupropion as part of a general practitioner (GP) management plan. Options for post-course support were participant-initiated only. The short course spanned 3 weeks and ran several times a year depending on need. Over a two-year period, five courses were attended by 32 participants, six of whom quit smoking (19%). It was inferred that cessation outcome was measured by self-report, however there was no information provided concerning at what time that occurred or whether there was longer-term follow-up.

### 4.4. Access-Promoting Elements of Interventions

The following categories emerged as a result of inductive analysis of elements of the interventions: workforce/organizational characteristics, cultural adaptations, support and follow-up, provision of instrumental support, self-determination/flexibility, and an integrative approach (Table 3). All elements within these categories served to promote acceptability and accessibility of interventions for the indigenous populations targeted.

#### 4.4.1. Cultural considerations

Elements of interventions depicting cultural tailoring were described in seven of the studies. Some elements reflect the importance of indigenous community input and ownership of interventions, including engaging in community consultation in planning and implementing the interventions [31,33,38–40] and conducting interventions in culturally-safe community settings, such as ACCHSs and other local indigenous-specific health service facilities [31,38–40]. Indigenous people, including health workers, facilitated groups, recruited, screened, and followed-up participants, or were otherwise involved in five of the interventions [31,33,35,38,39], while non-indigenous QL counselors received cultural sensitivity training in one study [34]. One study incorporated culturally-tailored resources in the form of culturally-specific flip charts, brochures, and course handouts [38].

#### 4.4.2. Workforce/organisation

The research team or health facility personnel and organizational support were noted in seven of the articles. In addition to the previously mentioned indigenous health worker-led programs, collaborative [33] and multidisciplinary teams [33,39,40] featured, as did referral to programs by health professionals [31,33,39]. Complementary workplace policies and management support to conduct or allow employees to attend programs [32,33,39] were described.

#### 4.4.3. Support and follow-up

Eight interventions were comprised of more than one face-to-face or counselor-initiated telephone counseling or support/course session [31,33–36,38–40]. Five articles described mechanisms for ongoing support beyond time parameters of the study protocol. Three of these studies described assessments of ongoing state or national telephone counseling support (QLs) [34–36] or an ongoing clinical service provided at an ACCHS [33]. Eight studies included follow-up ranging from 3–12 months for the purposes of outcome assessment of smoking status [31–36,38,40].

#### 4.4.4. Financial and transport assistance

Several of the articles reported providing instrumental support to enable access to these programs in the form of free or subsidized pharmacotherapy for a portion [38,39] or the duration of the intervention [32,33,35,40]. Three interventions noted transport to the intervention site was provided [33,38,39].

#### 4.4.5. Self-determination/flexibility

Six interventions allowed participants to refer themselves into the program rather than referral by health professional or meeting strict inclusion criteria [31–33,38,39]. Two interventions demonstrated flexibility by allowing participants to return for additional attempts to quit smoking during the duration of the programs [33,39]. Two interventions were described as having informal and interactive atmospheres within group sessions [38,39]. The QL interventions provided self-help materials to participants as well as the option to contact them when needed [34–36], with one study offering uninterrupted availability [35].

#### 4.4.6. Integrated approach

Six studies incorporated broader health and lifestyle support into the smoking cessation programs. Three studies required participants to attend a visit with a GP upon commencement of quit attempt [38–40]. Two Australian studies linked the smoking cessation program to government initiatives for indigenous people within the universal health coverage scheme [33,39], and one study linked participants to community resources [34]. Two Australian studies distributed behaviour modification items such as pedometers, water bottles, money boxes, relaxation tapes, and stress balls to reinforce positive health behaviours and adjunctive lifestyle modifications [33,39]. The role of stress in participants’ lives was recognized and management strategies discussed in one study [33]. Four studies described counseling interventions that were tailored to the needs of individual participants and included support and advice on a range of lifestyle issues [31,33,34,36]. Three studies measured expired carbon monoxide and/or volume and airflow upon inhalation and exhalation (spirometry) which provided participants with immediate visual information concerning lung health [31–33].

### 4.5. Discussion and Summary

We undertook this review with the aim of integrating information on interventions and describing elements to enhance engagement in and efficacy of individual-level interventions to assist indigenous people to quit smoking. No individual-level smoking cessation intervention studies involving indigenous populations from countries other than the four named were located. The complexity and costs of such therapeutic interventions may decrease availability of these services in developing countries [43]. In addition, the English language, peer-reviewed, and individual-level intervention inclusion criteria used in this review may have excluded reports of initiatives in other indigenous populations. Predominantly, the reviewed studies employed multi-component interpersonal interventions utilizing a form of counseling in combination with pharmacotherapy—an evidence-based method [44]. One counseling-only intervention [36] was included, although its authors acknowledged they had not assessed whether other support, such as NRT, was used by participants.

In this review, comparisons of cessation rates and assessments of intervention efficacy were complicated by different study designs, measurement intervals, cessation criteria, and multi-component programs. The only pharmacological aids used in these interventions were bupropion and NRT patches. Similar to results in other populations [45], Holt *et al.* [31] demonstrated that bupropion doubled cessation rates compared to placebo in Maori participants. Although comparison groups were not included in every study in this review, cessation rates in NRT interventions were generally higher than the 3–5% success rate of untreated quitters in other populations [46]. In fact, all forms of NRT have been found to increase the likelihood of successfully quitting by about 50–70% [47]. Trials of other pharmacotherapies (e.g., varenicline, other forms of NRT, or combinations of methods) have yet to appear in peer-reviewed literature describing interventions that report outcomes for indigenous populations, although one study by Richmond *et al.* showed promising results of using bupropion in conjunction with NRT in a group of prisoners, of whom 50% were Aboriginal Australians [48]. Furthermore, there is burgeoning evidence of a hierarchy of treatments with varenicline demonstrating superiority over bupropion, followed by NRT [49]. Ultimately, however, consideration of an individual’s preference and context should determine therapeutic use [50].

None of the interventions in this review assessed efficacy of pharmacotherapy alone, but rather most were multi-component; an evidence-based strategy to improve cessation rates [51]. Despite the absence of comparison groups in four of these studies, results appeared to confirm previous evidence that the combination of counseling, particularly multiple sessions, and medication is more effective for smoking cessation than either medication or counseling alone [51].

The strong dose-response relationship characteristic of clinical interventions [51] was evident in Ivers *et al.*’s [32] BI-only arm, but not in DiGiacomo *et al.*’s [33] high intensity intervention. Group formats have been shown to be more efficacious than less intensive interventions, however Mark *et al.*’s [38] group intervention reported a lower quit rate than Hayward *et al.*’s QL [36]. These contradictory findings highlight the complexities of evaluating multi-component interventions within diverse contexts.

Although seven interventions utilised NRT patches, just two assessed usage patterns and both reported poor adherence. Suboptimal use of NRT was considered related to participants sharing patches, not collecting additional supplies, or inadequate communication between practitioner and participant [32]. A range of contextual factors have been identified as barriers to medication adherence in Aboriginal Australians that are exacerbated by entrenched socio-economic differentials [52]. Inadequate dosing or adherence to NRT may produce symptoms that can be interpreted as side effects or futility of NRT, potentially inhibiting subsequent quit attempts [32]. Mark’s [38] group intervention participants had positive feelings towards another quit attempt, possibly highlighting the utility of higher intensity programs that can provide an extended period of support and discussion regarding quitting smoking. Higher intensity interventions and follow-up support have been shown to increase quit rates slightly [53].

Consistent with research in other populations [54], QLs appeared to be an effective and acceptable technique in aiding indigenous smokers to quit, despite the different approaches used and the absence of details on access and use of NRT patches. Caution must be exercised in interpreting these results however, given the self-report nature of follow-up smoking status and the criteria by which cessation was assessed. For example, Hayward *et al.* [36] defined 6-month prolonged abstinence as not having smoked on seven consecutive days or more than one day a week during two consecutive weeks, since their QL call; a definition based on, but not identical to recommendations of the Society for Research in Nicotine and Tobacco [55]. To be considered quit at the three-month follow-up, Boles *et al.* [35] and Maher *et al.* [34] used a 7-day point prevalence wherein participants had to report smoking ‘not at all’ and not having smoked for the past seven days. These assessments are not necessarily depicting continuous abstinence, or not having smoked since the quit date. Although the gold standard of cessation assessment is considered by many to be continuous abstinence, prolonged abstinence incorporates grace periods to accommodate lapses in cessation rather than counting these as failures [55].

Ivers *et al.* [32] and Holt *et al.* [31] were the only two studies that biochemically validated self-reported quit status. Although veracity of self-report has been questioned, it has been found to be accurate in most studies [56] and is a valid qualitative measure in Aboriginal Australians [57].

Another notable omission was that of peer/buddy support programs. Mark *et al.* provides one example of a support group, however other group programs were described as courses with didactic styles of information provision and no description of group interaction. Although there has been no consensus regarding whether group behavioural support programs are superior to individual models [58], the power of peer support and unity has been demonstrated in a case study of an indigenous community in Fiji that collectively decided to stop smoking. They used neither pharmacological nor counseling support, but rather enacted symbolic rituals and relied on their commitment to each other to strengthen their resolve, providing an inspiring example of the power of community [59].

Several of the reviewed articles offered insights regarding issues that arose during program implementation. These insights can inform future design and delivery of interventions, thereby underscoring the utility of publishing evaluations in peer-reviewed forums. For instance, Mark *et al.* [38] noted difficulties in following-up participants and the need to plan ahead for this potential challenge. Mark *et al.* [38] also highlighted the importance of addressing the combined use of marijuana with tobacco, as this can challenge quit attempts.

#### 4.5.1. Enhancing cultural appropriateness of interventions

Ensuring cultural appropriateness and acceptability of interventions is a recommended strategy in indigenous populations [37,60–62]. Elements identified in the review, although at times minimally described, included engaging in community consultation to ensure needs and preferences of the population are met, conducting interventions in culturally-safe, community-based settings, and ensuring community ownership of programs. Programs embedded within the culture’s philosophy of health and comprised of elements that reflect and respect the values of culture are likely to foster engagement of community members in interventions [63]. Likewise, holding interventions in community meeting places can facilitate participation and engagement [64]. Use of traditional cultural practices was demonstrated in two interventions, however these papers did not meet inclusion criteria of this review. These traditional practices included integration of Native Hawaiian and Western therapies in a community-based intervention in Hawaii [65] and a rapid inhalation ceremony and a tabu formalized through a kava ceremony in Fiji [59]. Additional studies reporting use of traditional practices may have been excluded due to the English language and peer-review inclusion criteria, however, they can assist in identifying acceptable and efficacious intervention elements.

The degree to which elements of interventions were perceived as culturally acceptable should be considered. Just one study reported no aspects of tailoring the intervention for Indigenous participants and did not provide free NRT [36]; factors that may have contributed to intervention efficacy. Group facilitators, counselors, health professionals or other project personnel in the majority of interventions were either from the cultural group or had undergone cultural sensitivity training. Collaborative multidisciplinary teams included AHWs who provided cultural mentorship. Culture-specific resources were used in an effort to tailor interventions [66]. Eliciting feedback from participants regarding intervention materials and delivery is a way to ensure acceptability of intervention content and communication style.

It is necessary to consider contextual factors which may impact on participants’ ability to engage in interventions. Cultural security can be achieved by not only acknowledging needs or preferences, but taking steps to address these needs in appropriate ways [67], via provision of instrumental support, for example. Although dispensing weekly allotments of NRT at repeated counselling sessions can facilitate a higher intensity intervention, transport and timing of intervention availability must be considered. Strategies such as providing cost-free pharmacotherapy and transportation to the intervention site may overcome these access and adherence barriers [52,68]. Ensuring a comfortable atmosphere, adopting a non-judgemental, non-intimidating interaction style in groups or individual sessions is likely to maintain engagement of participants, as are elements that demonstrate flexibility, participant choice, and control [69]. Flexibility in intervention format and implementation is particularly important in demonstrating appreciation of participants’ multiple competing priorities such as other health concerns, caregiving responsibilities, and stressful events [12].

Health disparities in indigenous peoples may reflect a lack of consideration of historical, social, and cultural contexts in the design and delivery of research and services [4,70]. Approaches to addressing high smoking rates should be relevant and appropriate to the needs and preferences of indigenous populations. To counter mistrust engendered by a history of colonization, relationships with indigenous communities should incorporate collaboration and mutually respectful partnership, including engaging with the community at all stages of the research process and enabling their meaningful involvement to ensure culturally safe practices [71,72]. Rather than impose non-indigenous perspectives and methods on Indigenous people, it is necessary to acknowledge and act with genuine consideration of their beliefs and cultures [11]. For example, the indigenous concept of health entails physical, mental, spiritual and emotional elements, reflecting both the individual and community, and is linked to political, economic, social and cultural aspects [4]. Strategies incorporating holistic approaches are more likely to promote engagement of indigenous people and be acceptable to them. Embracing this shift from mono-cultural health systems, that marginalize indigenous people, to intercultural health systems, will foster balance, reciprocity, and practice in which different cultures are valued and incorporated [4].

#### 4.5.2. Expanding perspectives of efficacy

High cessation rates are traditionally indicators of a ‘successful’ intervention and can impact continued funding of programs, however, other outcomes are important in establishing efficacy in indigenous populations. Given patient, provider, and system-related barriers to access, improving availability and acceptability of programs is an effective strategy to increase community engagement in smoking cessation and other health promotion programs. People from racial and ethnic groups have been found to use effective treatments less often and have lower success rates, despite wanting to quit [73]. Furthermore, people with higher stress levels and who live with smokers have lower abstinence rates [51]. Given that multiple quit attempts are indicators of eventual cessation [74], providing support for these quit attempts is likely to improve cessation rates in the long term. Enabling access further supports the normalization of quitting smoking as more individuals make quit attempts and diffuse the experience throughout the community. Efficacy may also be demonstrated by capacity built within the indigenous health workforce [75] and the development and strengthening of relationships with non-indigenous partners.

#### 4.5.3. A taxonomy for designing and reporting interventions

Publications identified by this review presented varying levels of description across content, implementation, personnel, and context of interventions. The reporting of multi-component cessation interventions was characterized by little detail regarding aspects of interventions and outcomes and resulted in several publications being excluded from review. Enhanced methodological description may highlight differential impacts of interventions [76] and ultimately help to eradicate inadequacies of policies and programs [4]. Greater consensus on describing intervention elements may be achieved through development of a taxonomy to categorise and compare programs and identify specific factors associated with effectiveness [77]. Krumholz [77] has developed a taxonomy to assess disease management programs which requires detail be provided across 8 domains: patient population; intervention recipient; intervention content; delivery personnel; method of communication; intensity and complexity of exposure and mix of program components; environment (context); and clinical outcomes. The use of a similar model in the future may assist in developing smoking cessation interventions via systematic reporting and analysis of programs to uncover the elements of effective interventions in indigenous populations.

#### 4.5.4. Which interventions show promise?

Despite low quit rates reported in some of the studies included in this review, they have revealed important cultural and contextual considerations for future design and delivery of individual-level smoking cessation interventions in indigenous populations. Among these factors are mode of delivery [35], addressing stress [33], the sharing of patches and other adherence issues [32], intensity of interventions including follow-up [32], cessation criteria, difficulties in achieving follow-up [38], cannabis use [38], and the importance of providing support (pharmacotherapy and counselling) without cost [38]. Previous research supports the use of multi-component strategies, particularly those that offer tailored counselling with pharmacotherapy (where possible, bupropion and varenicline should be utilised) without cost [51].

Interventions that incorporate elements to promote access and utilisation of support which can lead to increased cessation rates are critical. Effective treatments are rendered futile when they are inaccessible. No one intervention described in this review incorporated all access-promoting elements, as this is not feasible in most real-world situations. Indigenous populations are diverse and as such, interventions must be relevant, feasible, and acceptable to contexts and preferences.

## 5. Conclusions

Addressing the burden of smoking requires a multifaceted approach and large scale public health strategies including policy development. In addition, tailored and targeted approaches for indigenous populations are required, particularly for those who may not access population-based mainstream public health messages. The challenges for indigenous people are much greater and include poverty, marginalization, challenges in accessing resources, high rates of smoking, and acceptance of smoking in families and communities. This review has underscored the complexity of achieving smoking cessation and the need to collaboratively develop interventions that are acceptable and appropriate to local populations.

## Figures and Tables

**Table 1.** Proportion of indigenous and non-indigenous smokers by country.

**Country/Indigenous population**	**% of Indigenous people in population**	**% of Indigenous residents who smoke**	**% of non-Indigenous residents who smoke**
USA/Alaska Native and American Indian	1% ^[13]^	32% ^[14]^	22% ^[14]^
Australia/Aboriginal and Torres Strait Islander	2.3% ^[15]^	45% ^[16]^	20% ^[17]^
New Zealand/Maori	15% ^[18]^	45% ^[19]^	23% ^[19]^
Canada	3.3% ^[20]^		18% ^[21]^
First Nation, Metis, Inuit*		59% ^[22]^	
First Nation**		35.8% ^[23]^	
Metis**		33% ^[23]^	
Inuit**		59.8% ^[23]^	

* Living on a reservation;

** Not living on a reservation.

**Table 2.** Summary of intervention components.

**First Author (Year) Country**	**Study design/Setting**	**Intervention Format: G (Group) I-F (Individual-Face-to-face) I-P (Individual-Phone)**	**Pharmacotherapy: (NRT; Bupropion)**	**# indigenous participants/# indigenous participants followed-up**	**Contact Intensity**	**Cessation Outcome Assessment**	**Quit Rates**
**Holt (2005) NZ**	RCT/Public hospital Indigenous health unit	I-F and I-P	Bupropion	134 (88 Bupropion; 46 placebo)/78 (56 Bupropion; 22placebo)	6 clinic visits for re-assessments and counseling; follow-up telephone contact for re-assessment of smoking status up to 12 months following program	CA + CO at 3 months and 12 months	At 3 months—44% Bupropion group *vs.* 17% placebo (CO); At 12 months—21% Bupropion group *vs.* 10% placebo (CO)
**Ivers (2003) AU**	Pre-post/community health centres	I-F	NRT	111 (40 NRT; 71 BI-only)/93 (34 NRT; 59 BI-only)	One BI	At 6 months, PP of smoking status (undefined) + CO	15% BI + NRT quit (10% CO); 1% BI − only quit
**DiGiacomo (2007) AU**	Practice intervention/ACCHS	I-F	NRT	32/32	Unlimited weekly sessions (1/week)	CA at 6 months	9% remained smoke-free for 6 months
**Maher (2007) USA**	Pre-post survey/QL	I-P	NRT	101 completed follow-up survey	Calls initiated by QL counselor if participant set quit date on first contact; +4 calls if met criteria	7 day PP at 3 months	35% AI/AN; 31% for other races/ethnicities combined
**Boles (2009) USA**	Pre-post survey/QL	I-P	NRT	112 completed follow-up survey	Calls initiated by QL counselor if participant set quit date on first contact; ≤8 if met criteria	7 day PP at 3 months	22.2% Alaska Native; 40.7% non-Alaska Native
**Hayward (2007) CAN**	Pre-post survey/QL	I-P	NA	243 completed follow-up survey	Calls initiated by QL counselor ‘based on commitment to quit within a given timeframe’	At 6 months:7 day PP or 30 day PP; PA at 6 months	7 day PP: 18.9% Aboriginal; 16.5% non-Aboriginal; 30 day PP: 16.9% Aboriginal; 14.2% non-Aboriginal; 6 month PA: 10.7% Aboriginal; 8.8% non-Aboriginal
**Hensel (1995) USA**	Pre-post/CCHS	G	NRT	252/156 at 3 months; 111 at 6 months; 64 at 9 months; 24 at 12 months	4/6 sessions over a period of 2/7 weeks, respectively; F/U of smoking status 4 times over twelve months following course.	No longer smoking at 3, 6, 9, and 12 months	31% at 3 months; 30% at 6 months; 24% at 9 months; 21% at 12 months
**Mark (2004) AU**	Pre-post/community venue	G	NRT	115 completed pre-course survey/36 completed post-course/15 completed 3 month survey	4/6 sessions (1/week); 3 month telephone follow-up	24 hour PP at end of course; ‘Abstinence’ (undefined) at 3 months	44% (16 of 36 post-course survey completers) not smoking; 6% (15 of 115 pre-course survey completers) abstinent at 3 months
**Adams (2004) AU**	Pre-post/ACCHS	G and I-F	NRT	32/NA	2 3-hour sessions + 1 GP appt over 3 weeks	CA ‘to-date’ (2 years since course started; no F/U described)	19% quit smoking (n = 6)

**Legend**
NRT: Nicotine replacement therapy
RCT: Randomized controlled trial
CO: Validated with exhaled carbon monoxide measurement
BI: Brief intervention
AI/AN: American Indian/Alaska Native
ACCHS: Aboriginal Community Controlled Health Service
CCHS: Community Controlled Health Service
F/U: Follow-up
QL: QuitLine
NA: Not Applicable
GP: General Practitioner
**Cessation Outcome Assessments**
CA+CO: Continued abstinence—no cigarettes from target quit date validated by carbon monoxide measurement
CA: Continued abstinence—no cigarettes from quit date
PA: Prolonged abstinence—not having smoked on 7 consecutive days, or more than one day a week during 2 consecutive weeks
PP: Point prevalence—no smoking at all, not even a puff in specified time period (either 7 or 30 days).

**Table 3.** Access-promoting elements of interventions.

**Elements**	**Publications reporting use of element**
**Workforce**	
AHW involvement	*Adams (2006); Mark (2004); DiGiacomo (2007); Holt (2005); Boles (2009)*
AHW/project officer-led	*Adams (2006); Mark (2004)*
AHW model of successful attempt	*Adams (2006)*
Complementary workplace policy	*Adams (2006)*
Management support to run/attend	*Adams (2006); DiGiacomo (2007); Ivers (2003)*
Previous relationship between facilitator & community	*Adams (2006)*
Collaborative venture	*DiGiacomo (2007)*
Multi-disciplinary team approach	*Adams (2006); DiGiacomo (2007); Hensel (1995)*
Referral by health professionals	*Adams (2006); DiGiacomo (2007); Holt (2005)*
**Cultural Adaptations**	
Community-endorsed	*Adams (2006); Mark (2004); DiGiacomo (2007); Holt (2005)*
Community-based/culturally-safe setting	*Adams (2006); Mark (2004); DiGiacomo (2007); Holt (2005); Hensel (1995)*
Community consultation	*Mark (2004); DiGiacomo (2007); Holt (2005)*
Counselor trained in cultural sensitivity	*Maher (2007)*
Aboriginal-specific resources (video, flip charts, brochures, artwork)	*Mark (2004); Adams(2006)*
Advertised via ACCHS, AHWs, or GPs	*Mark (2004); DiGiacomo (2007); Holt (2005)*
**Support and follow-up**	
Ongoing support	*Adams (2006);DiGiacomo (2007);*
	*Maher (2007);Hayward(2010);Boles(2009)*
Follow-up contact for assessment	*Mark (2004); Ivers (2003); Holt (2005); Hensel (1995)*
**Instrumental support**	
Transport provided	*Adams (2006); Mark (2004); DiGiacomo (2007)*
No cost/subsidized	*Adams (2006); Mark (2004); DiGiacomo (2007); Ivers (2003); Boles (2009);*
	*Maher (2010); Hensel (1995)*
**Self-determination**	
Self-referral	*Adams (2006); Mark (2004); DiGiacomo (2007); Ivers (2003); Holt (2005)*
Informal/interactive atmosphere	*Adams (2006); Mark (2004)*
Not one-off/can try again	*Adams (2006); DiGiacomo (2007)*
**Integrated approach**	
Linked to community resources	*Maher (2007)*
Linked to Medicare initiatives	*Adams (2006); DiGiacomo (2007)*
General practitioner visit	*Adams (2006); Mark (2004); Hensel (1995)*
Behaviour modification items	*Adams (2006); DiGiacomo (2007)*
Motivational interviewing	*Maher (2007); Hayward (2007)*
Expired CO/spirometry	*Holt (2005); DiGiacomo (2007)*

Legend: AHW–Aboriginal Health Worker; NRT–Nicotine Replacement Therapy; CO–Carbon Monoxide; ACCHS–Aboriginal Community Controlled Health Service; GP–General Practitioner.
